# Proper Timing of Foot-and-Mouth Disease Vaccination of Piglets with Maternally Derived Antibodies Will Maximize Expected Protection Levels

**DOI:** 10.3389/fvets.2016.00052

**Published:** 2016-06-30

**Authors:** Aldo Dekker, Gilles Chénard, Norbert Stockhofe, Phaedra L. Eblé

**Affiliations:** ^1^Department of Virology, Central Veterinary Institute Lelystad, Wageningen UR, Lelystad, Netherlands; ^2^Boehringer Ingelheim Animal Health Operations BV, Weesp, Netherlands

**Keywords:** FMD, vaccine, maternal antibodies, porcine, timing of vaccination

## Abstract

We investigated to what extent maternally derived antibodies interfere with foot-and-mouth disease (FMD) vaccination in order to determine the factors that influence the correct vaccination for piglets. Groups of piglets with maternally derived antibodies were vaccinated at different time points following birth, and the antibody titers to FMD virus (FMDV) were measured using virus neutralization tests (VNT). We used 50 piglets from 5 sows that had been vaccinated 3 times intramuscularly in the neck during pregnancy with FMD vaccine containing strains of FMDV serotypes O, A, and Asia-1. Four groups of 10 piglets were vaccinated intramuscularly in the neck at 3, 5, 7, or 9 weeks of age using a monovalent Cedivac-FMD vaccine (serotype A TUR/14/98). One group of 10 piglets with maternally derived antibodies was not vaccinated, and another group of 10 piglets without maternally derived antibodies was vaccinated at 3 weeks of age and served as a control group. Sera samples were collected, and antibody titers were determined using VNT. In our study, the antibody responses of piglets with maternally derived antibodies vaccinated at 7 or 9 weeks of age were similar to the responses of piglets without maternally derived antibodies vaccinated at 3 weeks of age. The maternally derived antibody levels in piglets depended very strongly on the antibody titer in the sow, so the optimal time for vaccination of piglets will depend on the vaccination scheme and quality of vaccine used in the sows and should, therefore, be monitored and reviewed on regular basis in countries that use FMD prophylactic vaccination.

## Introduction

Foot-and-mouth disease (FMD) is a contagious disease of ruminants and pigs caused by FMD virus (FMDV). The disease is considered a major threat to commercially kept ruminants and pigs. As transmission of FMD occurs even when animal movement is prohibited, the major transmission routes most likely include people moving between farms. “Stamping out” in a small radius around infected farms has recently been applied in several outbreaks, but this involves many people moving between potentially infected farms. Therefore, a control measure that requires fewer people, such as vaccination, is preferred. Furthermore, from an ethical point of view, vaccination is preferred to stamping out farms at risk (1). However, maternally derived antibodies can interfere with the development of vaccine-induced immunity (2, 3). There has been discussion whether FMDV oil vaccines in pigs can induce immunity irrespective of maternally derived antibodies but Francis and Black (4) showed that maternally derived antibodies hinder the development of protective immunity. In cattle, it has been shown that a heterologous strain within the same serotype can induce an immune response in calves with maternally derived antibodies (5), so the immune response is not necessarily blocked by maternally derived antibodies. In addition, in pigs with maternally derived antibodies, a response to influenza vaccination can also be measured in the presence of maternally derived antibodies. However, the response is lower and will probably not protect (6). One of the options to boost immunity levels is repeated vaccination, i.e., first vaccination in the presence of maternally derived antibodies, to prime the immune system, and a second vaccination 1 or 2 months later. However, the costs of two vaccine administrations are high, not only due to the cost of vaccine but also the logistics and labor costs, which are often higher. Therefore, it may be preferable to optimize the timing of a single vaccination.

The objective of this study was to determine the factors that influence the optimal age for FMDV vaccination of piglets. We measured the neutralizing antibody response in piglets born to vaccinated sows at 3, 5, 7, and 9 weeks of age. The neutralizing antibody titer was compared with non-vaccinated piglets from the same sows, as well as with vaccinated piglets born from non-immune sows.

## Materials and Methods

### Vaccine

The antigens used in the vaccines in this study were produced on an industrial scale using baby hamster kidney (BHK) cells. The antigens were inactivated with binary ethyleneimine (BEI) and concentrated approximately 100 times by two cycles of polyethylene glycol (PEG) precipitation. The antigen concentration was determined by sucrose gradient analysis (7). The oil vaccines were formulated using a mineral oil as adjuvant in a double oil emulsion, as previously described (8). The vaccines were formulated to contain at least six PD_50_ per dose (i.e., six times the dose that protects 50% of the cattle against virulent challenge in the tongue). One trivalent vaccine batch was used for the sows and one monovalent vaccine batch was used for the piglets. A single dose was 2 ml.

### Vaccination of Sows

The sows (SPF pigs TN20 and TN70 from the genetics company Topigs Norsvin) used in this study were available from a vaccine safety test. The sows had not been vaccinated against FMD before beginning the study and were free of antibodies against FMDV. The sows were vaccinated intramuscularly with trivalent FMDV vaccine containing O Manisa, Asia-1 Shamir, and A TUR/14/98 antigen. The sows were vaccinated at day 36, 57, and 85 of gestation. Piglets were born after 112–114 days of gestation.

### Vaccination Piglets

A total of five vaccinated sows were selected that had nine or more piglets. From each sow, two piglets with maternally derived antibodies were selected randomly and assigned to one of the five groups of piglets (except in Group 5 where one sow supplied three piglets and one sow only one piglet). Two non-vaccinated sows supplied each five piglets for Group 6 (vaccinated piglets without maternally derived antibodies).

Piglets with maternally derived antibodies in Groups 1, 2, 3, and 4 were vaccinated intramuscularly with a single dose of monovalent FMD vaccine containing A TUR/14/98 at 3, 5, 7, and 9 weeks of age, respectively. The piglets that were used as vaccination control (Group 6) were vaccinated at 3 weeks of age. Serum samples were collected weekly up to 6 weeks after vaccination.

### Virus Neutralization Test

Sera were tested for virus neutralizing antibodies against FMDV A TUR/14/98, O Manisa, and Asia-1 Shamir, using primary porcine kidney cells (9). Twofold dilutions of the serum samples were tested starting with undiluted serum. Titers were expressed as log_10_ of the reciprocal of the dilution that inhibited virus growth in 50% of the wells. For calculation of the mean titers and for the use in statistical tests, we used 0 for the observations with a log_10_ titer of <0.30.

### Statistical Analysis

Because the same animals were sampled at different time points, we analyzed the antibody response in the sows by forward selection in a linear mixed-effects model (10, 11) using the virus neutralization tests (VNT) titer as continuous response variable, the time after vaccination and strains as possible explanatory nominal variables. The animal ID was included as random variable (model M1). The relation between neutralizing antibody titer in the sows 3 weeks after the last vaccination (approximately 30 weeks of gestation), and the antibody titer in the piglets just after colostrum uptake was analyzed by linear regression. Using forward selection, the effect of serotype was analyzed (M2).

The median time for which neutralizing antibody titers in the piglets were detected (titer ≥0.3) was analyzed in a logistic mixed-effects model (10, 11). Whether or not a neutralizing antibody titer was observed was the binary result variable. Possible nominal explanatory variables used were group, serotype, and mother, whereas the possible continuous explanatory variables were age and neutralizing antibody titer at birth. The piglet was entered as random variable (M3).

The half-life of the neutralizing antibody titers was analyzed in a linear mixed-effects model (10, 11). The neutralizing antibody titer measured was the result variable. Possible nominal explanatory variables used were group, serotype, and mother, whereas the possible continuous explanatory variables were age, neutralizing antibody titer of the dam, and neutralizing antibody titer at birth. The piglet was entered as random variable (M4). In the abovementioned analyses, the best fitting model was selected in a forward selection procedure using the Aikaike information criterion (12, 13). Analysis was performed using R version 3.2.3 and lme4 library version 1.1.10 using default settings.

## Results

**Figure 1:**
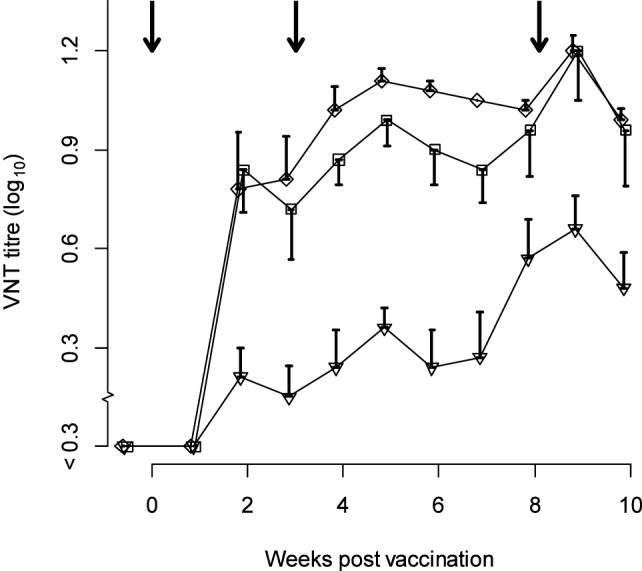
**Titers observed in sows (*n* = 5) vaccinated with trivalent FMD vaccine (at least six PD_50_ per dose) three times during gestation**. Antigens included in the vaccine were type A TUR/14/98 (diamonds), O Manisa (squares), and Asia-1 Shamir (triangles). The arrows indicate the time of vaccination. The error bars (only one direction to avoid overlap) indicate the SEM.

The neutralization titers observed in the sows are given in Figure 1. The linear mixed-effects model (M1, Table 1) showed that the neutralizing antibody response was significantly higher 2–10 weeks after vaccination compared with the titer at the time of vaccination, but differed significantly between strains. The titer induced by serotype Asia-1 was approximately 0.5 log_10_ lower than the titers against A TUR/14/98 and O Manisa (Table 1).

**Table 1:** **Final selected linear mixed-effects model (M1) using the VNT titer in the sows (*n* = 5) as continuous response variable, the time after vaccination, and strains as possible explanatory nominal variables**.

Random effects	Variance	SD	
Sow	0.007	0.08	
Residual	0.05	0.23	

**Fixed effects**	**Estimate**	**SE**	***t*-Value**

Intercept	−0.3	0.07	−4.5
0.9 weeks post vaccination	0.0	0.08	0.0
1.9 weeks postvaccination	0.6	0.08	7.4
2.9 weeks postvaccination	0.6	0.08	6.8
3.9 weeks postvaccination	0.7	0.08	8.6
4.9 weeks postvaccination	0.8	0.08	9.9
5.9 weeks postvaccination	0.7	0.08	9.0
6.9 weeks postvaccination	0.7	0.08	8.7
7.9 weeks postvaccination	0.9	0.08	10.3
8.9 weeks postvaccination	1.0	0.08	12.4
9.9 weeks postvaccination	0.8	0.08	9.8
Strain A TUR/14/98	0.5	0.04	12.4
Strain O Manisa	0.5	0.04	10.8

*The animal was included as random variable*.

Table 2 shows the neutralizing antibody titers against A TUR/14/98 in the piglets in the various groups. In the piglets with maternally derived antibodies (Groups 1–5), the mean neutralizing antibody titer (log_10_) against A TUR/14/98 was 1.7 (SEM 0.05) at birth. The mean neutralizing antibody titer (log_10_) against O Manisa was slightly higher (mean, 2.0 SEM 0.06) and lower for serotype Asia-1 Shamir (mean, 1.3 SEM 0.06). The neutralizing antibody titer of the piglets was strongly correlated with the neutralizing antibody titer of the sows. On average, a 1 log_10_ higher antibody titer in the sows resulted in a 1 log_10_ higher antibody titer in the piglets (univariate linear regression, data not shown). However, the relation was different for each serotype, and an interaction effect was found between serotype and the neutralization titer of the dam (M2, Table 3). The interaction effect was caused by the fact that the titers in the dam for type A TUR/14/98 were 0.9 or 1.05, and no relation between antibody titer in the dam and the piglet for this serotype could be determined (Table 3).

**Table 2:** **Age and neutralizing antibody titer against the strain used for vaccination (A TUR/14/98)**.

**Group**	**Number**	**Age of vaccination (days)**			**VNT titer (A TUR/14/98) at birth**			**VNT titer (A TUR/14/98) at vaccination**		
		**Minimum**	**Mean**	**Maximum**	**Minimum**	**Mean**	**Maximum**	**Minimum**	**Mean**	**Maximum**
1	10	21	22	23	0.90	1.7	2.10	0.60	1.2	1.65
2	10	35	36	37	1.35	1.8	2.25	0.45	0.9	1.50
3	10	49	50	51	0.90	1.7	2.25	<0.30	0.5	1.05
4	10	63	64	65	1.35	1.8	2.40	<0.30	0.1	0.60
5	10	NA	NA	NA	1.35	1.8	2.25	NA	NA	NA
6	10	20	21	21	<0.3	<0.3	<0.3	<0.3	<0.3	<0.3

*Mean titers are calculated considering <0.30 as 0*.

**Table 3:** **Final selected linear model (M2) using the VNT titer at birth of the piglets (*n* = 50) as continuous response variable, the VNT titer of the dam and strains as possible explanatory variables**.

	**Estimate**	**SE**	***t*-Value**	***p*-Value**
Intercept	0.6	0.1	5.5	≪0.001
VNT titer of dam	1.6	0.2	8.3	≪0.001
Strain A TUR/14/98	1.3	0.6	2.2	0.03
Strain O Manisa	0.6	0.2	3.5	0.0006
Interaction VNT titer dam and strain A TUR/14/98	−1.7	0.6	−2.9	0.005
Interaction VNT titer dam and strain O Manisa	−0.7	0.2	3.2	0.001

*Strain Asia-1 was the baseline variable*.

**Figure 2:**
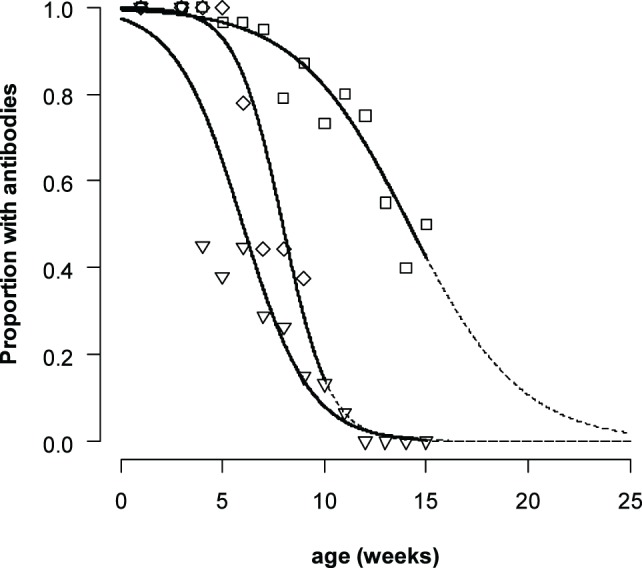
**Proportion of piglets with FMDV neutralizing antibody titers ≥0.3**. For non-vaccinated piglets A TUR/14/98 (only Group 5, diamonds, central line). For all piglets with maternally derived antibodies (Group 1–5) for serotype O Manisa (squares, line on the right) and serotype Asia-1 Shamir (triangles, line on the left). The data were analyzed by a logistic mixed-effects model. The dashed line indicates the extrapolated part of the curve.

The duration for which maternally derived antibodies could be detected differed for the different serotypes (Figure 2). The logistic mixed-effects model (M3, Table 4) showed that the presence of VNT titres was statistically dependent on the titer at birth, age, the serotype with an interaction effect between age, and serotype. The median time that antibodies are present increases with approximately 29–53 days when the titer at birth is 1 log_10_ higher, depending on the serotype. For piglets with a neutralizing antibody titer of 2 log_10_ at birth (the mean titer for O Manisa in the dataset), the model produced a median time that neutralizing antibodies could be detected of 9, 14, and 9 weeks, respectively, for serotype A TUR/14/98, O Manisa, and Asia-1 Shamir. Interestingly, when the analysis was performed with only the non-vaccinated piglets (Group 5), a significant interaction effect between age and serotype was found, indicating that the median time for which antibodies are present are different for different serotypes even if the piglets were born from the same mother and started with the same neutralizing antibody titer at birth (results not shown).

**Table 4:** **Final selected logistic mixed-effects model (M3) using the presence of a VNT titer (<0.3 is negative, 0.3 or higher is positive) in the piglets (*n* = 50) as binary response variable, the age (days), the VNT titer at birth, and strains as possible explanatory variables**.

Random effects	Variance	SD		
Piglet	2.7	1.7		

**Fixed effects**	**Estimate**	**SE**	***z*-Value**	***p*-Value**

Intercept	−0.2	0.9	−4.5	0.8
VNT titer at birth	6.5	0.8	8.1	≪0.001
Age (days)	−0.2	0.02	−8.8	≪0.001
Strain A TUR/14/98	2.7	2.6	1.0	0.3
Strain O Manisa	−1.2	1.2	−0.9	0.3
Interaction age and strain A TUR/14/98	−0.4	0.05	−0.8	0.4
Interaction age and strain O Manisa	0.1	0.02	3.6	0.0003

*The piglet was included as random variable. Strain Asia-1 was the baseline variable*.

The total number of sera tested in Groups 1–5 was 421; the number of left censored data (sera with a titer <0.3) was different for each serotype 57 for O Manisa, 71 for A TUR/14/98, and 254 for Asia-1. However, none of the piglets had a titer <0.3 at the beginning of the study. In the linear mixed-effects model, to study of the decline of neutralizing antibody titers in the piglets (M4, Table 5), only sera with a titer of 0.3 or higher were included. The analysis showed that the antibody titer depended on the age of the piglets, the titer of birth, the titer of the dam, and the serotype. There were interaction effects found between serotype and age, titer at birth and age, and titer of the sow and serotype. The interaction effect between both serotype and titer at birth with age shows that half-life of neutralizing antibodies detected in our study depends on the serotype and the titer at birth. The estimated half-life of neutralizing antibodies for a piglet that started with a neutralizing antibody titer of 2 log_10_ (based on the observed mean titer in week 1 for O Manisa) was 11, 16, and 12 days for, respectively, serotype A TUR/14/98, O Manisa, and Asia-1.

**Table 5:** **Final selected linear mixed-effects model (M4) using the VNT titer in the piglets (*n* = 50) as continuous response variable, the age, VNT titer at birth, VNT titer of the sows, and strains as possible explanatory variables**.

Random effects	Variance	SD	
Piglet	0.01	0.10	
Residual	0.04	0.20	

**Fixed effects**	**Estimate**	**SE**	***t*-Value**

Intercept	0.2	0.07	2.8
Age (days)	−0.02	0.002	−8.3
VNT titer at birth	0.8	0.06	12
VNT titer dam	0.4	0.1	3.9
Strain A TUR/14/98	−0.8	0.4	−2.0
Strain O Manisa	−0.05	7	−0.8
Interaction age and strain A TUR/14/98	−0.002	0.002	−0.8
Interaction age and strain O Manisa	0.006	0.001	6.4
Interaction age and VNT titer at birth	−0.005	0.001	−5.3
Interaction VNT titer dam and strain A TUR/14/98	0.8	0.4	1.9
Interaction VNT titer dam and strain O Manisa	−0.1	0.09	−1.6

*The piglet was included as random variable. Strain Asia-1 was the baseline variable*.

**Figure 3:**
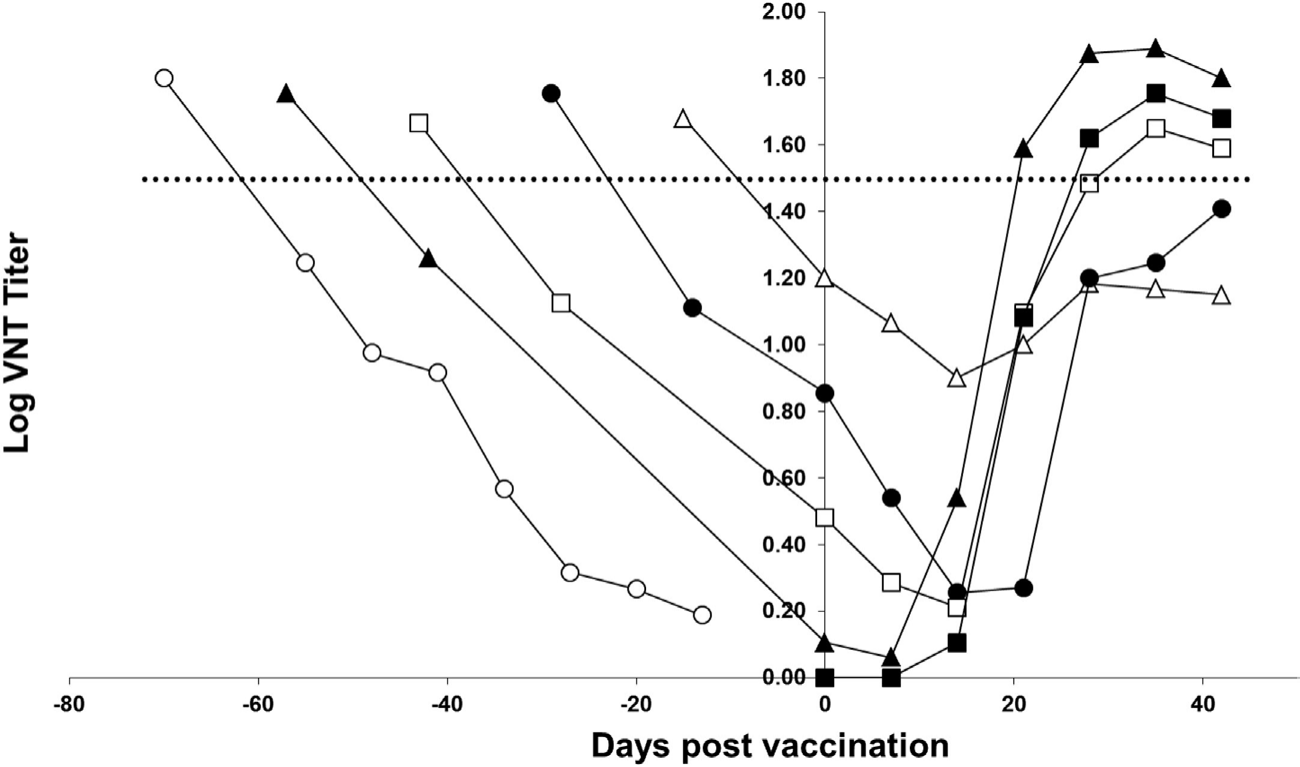
**FMD type A TUR/14/98 serology profile following homologous vaccination of piglets with maternally derived antibodies**. Piglets were either vaccinated at 3 (open triangle), 5 (solid circle), 7 (open box), or 9 (solid triangle) weeks of age. One group of piglets (open circle) did not receive a vaccination and one group did not have maternally derived antibodies (solid box). The horizontal line indicates the mean virus neutralization titer observed 28 days postvaccination in a previous study in pigs vaccinated with a vaccine containing six PD_50_ per dose.

Figure 3 shows the response of the piglets to homologous vaccination with a monovalent FMDV serotype A TUR/14/98 vaccine. The curves show that all groups of piglets respond to vaccination; after vaccination, the curve does not follow the decrease of maternally derived antibodies observed in non-vaccinated piglets. However, piglets vaccinated 4 or 5 weeks after birth did not have a response to the vaccine up to the level seen in a previous study (horizontal dotted line in Figure 3) in which pigs were vaccinated with an FMD vaccine containing six PD_50_ per dose. The response in the piglets vaccinated 7 and 9 weeks after birth was similar to the response observed in pigs vaccinated with an FMD vaccine containing six PD_50_ per dose. Based on this six PD_50_ per dose threshold value, the response in the piglets vaccinated 7 and 9 weeks after birth was deemed sufficient, and the piglets vaccinated 9 weeks after birth had an earlier and initially a higher response than the piglets born from non-vaccinated sows.

## Discussion

The objective of this study was to determine the optimal age for FMD vaccination of piglets based on measurement of serological titers following vaccination and the corresponding level of protection that can be expected. In contrast to an earlier publication (4), we observed a response in piglets with maternally derived antibodies vaccinated 3 weeks after birth; following vaccination, the curve did not follow the decrease of maternally derived antibodies observed in non-vaccinated piglets. In our experiment, when considering the six PD_50_ per dose threshold value (shown in Figure 3) the response to vaccination was sufficient when piglets were vaccinated 7–9 weeks after birth. Piglets can respond to vaccination in the presence of maternally derived antibodies, as has been shown before for FMDV vaccines (14), as well as for influenza vaccines (6). Our experiment confirms the earlier finding that the higher the maternally derived antibody titer, the lower the response to vaccination. To induce a neutralizing antibody titer likely to confer protection, piglets should ideally be vaccinated when maternally derived antibodies are at very low level. The median time that maternally derived antibodies are present in piglets depends on the titer at birth and the serotype. The titer at birth depends on the titer of the sows and the serotype of the vaccine. We observed a large variation in antibody titers between seroptypes in the sows, which, in turn, resulted in differences between serotypes in maternal antibody titers and, consequently, the optimal time for vaccination of the piglets. The antibody response for Asia-1 Shamir in pigs was known to be lower in comparison to A TUR/14/98 and O Manisa (15). It is difficult to extrapolate our findings to other FMD vaccines since different vaccine formulations might induce higher or lower responses in sows. The vaccination protocol of the sows can also influence the outcome. In our case, the sows were vaccinated three times during pregnancy, because the study was a repeat-dose safety study for a vaccine marketing authorization application. In a field situation, it is probably easier to vaccinate sows one or two times per year, which will probably result in higher variation in titers in the piglets compared with a scheme suggested earlier (16), where sows are vaccinated before pregnancy and boosted once during pregnancy to obtain the highest titers in the piglets. Therefore, it is important that authorities responsible for vaccination monitor the response and study the optimal time for vaccination on a regular basis, as different FMD vaccines used in sows can influence the immune status of the mother.

A remarkable finding in both the logistic mixed-effects model and the linear mixed-effects model was the interaction effect between age and serotype. In the linear mixed-effects model, this interaction could be explained in the difference in censoring for the different serotypes; for serotype Asia-1, there were more observations with a VNT titer <0.3. In the logistic mixed-effects model, there was no censoring. This indicates that the decrease of maternally derived antibodies was different between the different serotypes. Such an interaction effect was not observed when we studied the duration of maternally derived antibodies in calves (5). The difference can be explained by the fact that decrease of maternally derived antibodies is not only due to antibody metabolism but also due to the growth of the piglet (3), in combination with the fact that the titers at birth were not the same. The biggest change in weight (relatively) is in the beginning, when piglets grow from 1–2 kg to approximately 10 kg within a month. This is almost a 10-fold increase in weight. The next 10-fold increase takes more than 5 months. In the profile of the O Manisa, maternally derived antibody titers, approximately a 1 log_10_ decrease is seen in the first 4 weeks, then the decrease becomes less steep. For serotype Asia-1 and the non-vaccinated piglets for A TUR/14/98, a 1 log_10_ decrease is also observed in the same period. But for serotype Asia-1, most piglets have titers below the detection level of the assay after 4 weeks (Figure 2), so it is not possible to assess the reduction in slope. It is unlikely that the metabolization of maternally derived antibodies is different between the different serotypes.

The antibody response in this study was only followed for 6 weeks after vaccination, but fattening pigs should be protected for at least 6 months. Therefore, it is advisable that further studies in countries using vaccination are carried out, also to obtain data on antibody response in a field situation.

The most important result from our study was the observation that large differences arise in the duration of the maternally derived antibodies, which mainly depend on the titer at birth, which, in turn, depends on the titer in the sows. Therefore, every country that uses vaccination to control FMD in swine populations should determine the optimal vaccination strategy for the vaccine they are using monitoring titers in sows. A reassessment of the strategy is warranted when new FMD vaccines are introduced.

## Ethics Statement

Study approved by the Central Veterinary Institute, Dier experimenten commissie (Animal Ethics Committee).

## Author Contributions

All authors contributed to the design of the experiment and analysis of the data, and reporting was performed by the first two authors.

## Conflict of Interest Statement

The authors declare that the research was conducted in the absence of any commercial or financial relationships that could be construed as a potential conflict of interest.
